# Isolation and characterisation of house dust mite and formulation of its extract into sub lingual tablets for sub lingual immuno therapy

**DOI:** 10.1186/2045-7022-4-S2-P2

**Published:** 2014-03-17

**Authors:** Paranjothy Kanni, Venkat Ramana Dhurva, Nagendraprasad Komarla, Vishwanath Boguda

**Affiliations:** 1 Bangalore Allergy center, 6A 36th Cross , 28th main, 9th block , Jayanagar, Bangalore, 560069, India; 2 Aditya Bangalore Institute of Pharmacy education and research, Pharmaceutics, Bangalore, India; 3 Bangalore Allergy center, yashaha laboratories, Bangalore, India

## BACKGROUND

The house dust mites are cosmopolitan guest in human habitation. Dust mites occur in nearly all homes. They are generally found in pillows, bed sheets, mattress, stuff toys, carpets etc. Dust mites feed on organic detritus such as flakes of shed human skin and flourish in stable environment of dwellings. Exposure to house dust mites is recognized as major cause allergy worldwide. The European house dust mite (Dermatophagoides Pteronyssinus) and the American house dust mite (Dermatophagoides Farina) are two major species responsible for allergy.

## METHOD

The objective of the current study is to design, develop and optimize a sub lingual tablets by isolation and characterization of House Dust Mite and formulation into Sub lingual tablets with its purified extract for sublingual immunotherapy. Drug delivery via sub lingual mucous membrane is considered to be a promising alternative to the subcutaneous route .The drug absorbed via sub lingual blood vessels bypasses the hepatic first-pass metabolic processes giving acceptable bio availability with low doses and hence decreases the side effects. Sub lingual tablets containing house dust mite extract were prepared by wet granulation method using different ingredients such as Croscaremellose sodium, Crospovidone, Sodium starch glycolate , Starch, Sucralose ,Sodium saccharin, Mannitol, Microcrystalline cellulose, Talc and Magnesium stearate.

## RESULTS

The compatibility studies of the polymers with drug was checked by FT-IR spectroscopy. The FT-IR spectra revealed that, there was no interaction between polymers and drug. Polymers used were compatible with drug. The granules were evaluated for their physical properties like angle of repose, bulk density, compressibility index and Hausner's ratio and were found to be satisfactory. The Hardness, Weight variation, Thickness, Friability and Drug content of the tablets were found to be acceptable according to pharmacopoeial limits. Accelerated stability studies at 40ºC/ 75% RH of this optimized formulation indicated no appreciable change.The results lead to the following Specification : 6mm flat tablets with beveled edges with Disintegration time of 20±5 seconds, Assay of 100±15% of protein Nitrogen Units of the stated label claim and Dissolution of not less than 95% in 5 minutes.

## CONCLUTION

An optimized formulation of Sub lingual tablets of House Dust Mite with all desirable characteristics and parameters were developed and evaluated.

